# A review of structural neuroimaging in schizophrenia: from connectivity to connectomics

**DOI:** 10.3389/fnhum.2014.00653

**Published:** 2014-08-25

**Authors:** Anne L. Wheeler, Aristotle N. Voineskos

**Affiliations:** ^1^Kimel Family Translational Imaging Genetics Laboratory, Centre for Addiction and Mental Health, Research Imaging CentreToronto, ON, Canada; ^2^Department of Psychiatry, University of TorontoToronto, ON, Canada

**Keywords:** magnetic resonance imaging, diffusion tensor imaging, schizophrenia, connectomics, connectivity, white matter

## Abstract

In patients with schizophrenia neuroimaging studies have revealed global differences with some brain regions showing focal abnormalities. Examining neurocircuitry, diffusion-weighted imaging studies have identified altered structural integrity of white matter in frontal and temporal brain regions and tracts such as the cingulum bundles, uncinate fasciculi, internal capsules and corpus callosum associated with the illness. Furthermore, structural co-variance analyses have revealed altered structural relationships among regional morphology in the thalamus, frontal, temporal and parietal cortices in schizophrenia patients. The distributed nature of these abnormalities in schizophrenia suggests that multiple brain circuits are impaired, a neural feature that may be better addressed with network level analyses. However, even with the advent of these newer analyses, a large amount of variability in findings remains, likely partially due to the considerable heterogeneity present in this disorder.

## Introduction

Schizophrenia affects approximately 1% of the population and is characterized by disordered thought processes as well as impaired emotional responses. The underlying neural substrates of schizophrenia likely involve alterations of brain circuitry. *In vivo* neuroimaging approaches have made significant inroads toward the description of these brain abnormalities though variability in results has precluded consensus on precise neural correlates of the illness. The majority of neuroimaging studies examine the brains of chronic patients who have lived with schizophrenia for years. What cannot be known from these results is the timing of brain changes: were they present before the onset of the disease, or did they manifest over the course of the illness? These two hypotheses are not mutually exclusive, but cannot be teased apart in studies that examine brain structure and function in chronic patients. Additionally, the vast majority of patients in these studies are taking antipsychotic medications to treat the symptoms of the illness and the effects of long-term medication exposure on brain structure could confound interpretation of these results. The examination of patients who are early in the course of the illness (first episode) and often have not been treated with medication, as well as groups that are at a high risk for developing the disorder (high-risk and family studies), aim to address these issues. Moreover, understanding brain abnormalities in these groups may make it possible to identify vulnerability early and allow for interventions to help prevent or delay progression to chronic illness.

As distributed structural circuits of cortical and subcortical areas serve normal brain functions, disrupted communication within and between brain regions may be the core pathology of schizophrenia. Two decades ago it was proposed that the symptoms of schizophrenia were due to alterations in cerebral connectivity (Friston and Frith, 1995), a hypothesis that has been gaining traction in recent years with the availability of advanced imaging techniques that can be used to address this theory (Kochunov and Hong, 2014). White matter in the brain consists of the axonal projections to other neurons and brain areas and forms the basis for connectivity in the brain. Structural integrity of white matter can be examined indirectly with diffusion-weighted imaging. Alternatively, computing inter-regional correlations of regional gray matter morphology (structural co-variance) can assess coupling of brain regions, an index of connectivity (Alexander-Bloch et al., 2013). The application of complex network analysis methods has aided in the description of altered connectivity in the brains of schizophrenia patients. Widespread variability of results exists in structural connectivity studies in all types of schizophrenia patients and high-risk subjects. Several factors likely contribute to these inconsistencies in findings including methodological differences between studies, variability in the clinical presentation of schizophrenia, differences in causal mechanisms underlying schizophrenia and variation in moderator variables across studies.

## Diffusion weighted imaging studies

Diffusion tensor imaging (DTI), a diffusion-weighted MRI method, assesses the diffusion of water molecules in order to map microscopic details about white matter fiber structure. It is based on the principle that water moves most easily along axonal bundles because there is the smallest number of obstacles to prevent movement in this direction. Diffusion measurements along different axes are fitted to a 3D ellipsoid (tensor) at each voxel. The most commonly assessed metric in DTI studies is fractional anisotropy (FA). FA reflects the magnitude and directionality of water diffusion. Specifically, FA represents the fraction of the tensor that can be assigned to diffusion that is constrained along one axis only, known as anisotropic diffusion. FA values are scaled from 0 (isotropic) to 1 (anisotropic). FA and other DTI metrics are thought to reflect the microstructural properties of white matter tracts, including abnormal coherence of the fiber tracts, fiber density, axonal diameter, and myelination, although the exact microstructural correlates are not clear. Different methods are available to analyze DTI scans. Tractography allows probable trajectories of fiber tracts to be calculated and visualized with an index of fiber integrity averaged over the reconstructed tract, allowing tract-specific measurements. In voxel-based morphometry (VBM) structural images are spatially normalized onto a stereotaxic brain atlas, which allows DTI metrics to be calculated on a voxel by voxel basis. Statistical comparison can then be made between populations, which can be applied to the whole brain or specific regions of interest (ROIs). More recently, tract-based spatial statistics (TBSS) has been developed in which voxel-based diffusion metrics are projected onto an alignment-invariant tract representation (Smith et al., 2006). DTI is a relatively new technique and many of the early studies summarized here evaluate relatively small groups of mainly chronic schizophrenia patients.

### Chronic patients

**Figure 1:**
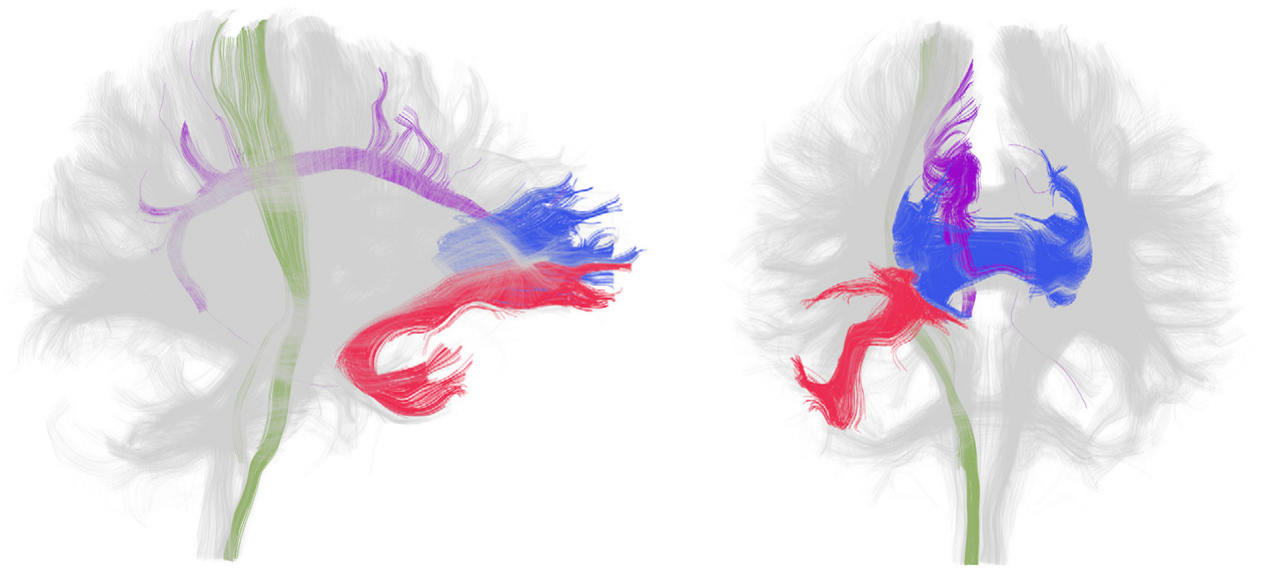
**White matter tracts most frequently identified as disrupted in patients with chronic schizophrenia**. Lateral (left) and frontal (right) view of whole brain tractography as identified with a clustering method (Voineskos et al., 2009) and visualized in the 3D Slicer program in a sample subject. The uncinate fasciculus (red), cingulum bundle (purple), corpus callosum (blue, only the genu is shown) and internal capsule (green, only a portion spanning from the corticospinal tract is shown) are displayed in color and the rest of the tracts are gray. Note that disruption in these tracts has been shown bilaterally but association and projection tracts are only colored in the right hemisphere for clarity.

In patients with chronic schizophrenia, decreased FA within prefrontal and temporal lobes, as well as abnormalities within the fiber bundles connecting these regions, particularly the uncinate fasciculus and the cingulum bundle as well as the corpus callosum and internal capsule, are the most frequent positive findings (Figure 1). An earlier review of the literature reported that white matter in the frontal and temporal lobes were the most frequently reported areas showing reduced FA in DTI studies of schizophrenia (Pettersson-Yeo et al., 2011). More recent studies support this with VBM (Walther et al., 2011) and TBSS (Scheel et al., 2012; Roalf et al., 2013; Fujino et al., 2014) analyses reporting decreased FA in frontal and temporal regions, as well as in the parietal and occipital cortices. Moreover, a meta analysis of 15 voxel based DTI studies in schizophrenia determined that significant reductions were present in two regions: the left frontal deep white matter and the left temporal deep white matter (Ellison-Wright and Bullmore, 2009). Reduced FA has also been described in superficial white matter in the left posterior parieto-occipital cortex and the left frontal lobe (Nazeri et al., 2013). White matter tracts interconnecting the frontal lobe, thalamus and cingulate gyrus traverse the frontal lobe and tracts interconnecting the frontal lobe, insula, hippocampus-amygdala, temporal and occipital lobes traverse the temporal lobe.

A reduction in FA has been shown in specific association, commissural and projection white matter tracts in chronic patients with schizophrenia (Table 1). Diffusion abnormalities identified in the arcuate fasciculus are predominantly in the left hemisphere though two studies have reported increased, rather than decreased, FA in this tract in schizophrenia patients relative to control subjects (Rotarska-Jagiela et al., 2009; Knochel et al., 2012). The corpus callosum is the largest white matter structure in the brain and is the main commissural tract as it connects the two hemispheres. Some studies find anisotropy differences in schizophrenia patients restricted to the anterior portion that connects the left and right prefrontal cortex, known as the genu (Hubl et al., 2004; Shergill et al., 2007; Kubicki et al., 2008).

**Table 1:** **White matter tract alterations in patients with chronic schizophrenia**.

**White matter tract**	**Method**	**References**
Uncinate fasciculus	VBM	Burns et al., 2003; Hubl et al., 2004; Mori et al., 2007; Munoz Maniega et al., 2008; Sussmann et al., 2009
	TBSS	Seal et al., 2008; Knochel et al., 2012
	Tractography	Voineskos et al., 2010; de Weijer et al., 2011; Kunimatsu et al., 2012
Cingulum bundle	VBM	Kubicki et al., 2003, 2005; Sun et al., 2003; Wang et al., 2004; Fujiwara et al., 2007; Mori et al., 2007; Skelly et al., 2008; Segal et al., 2010
	TBSS	Camchong et al., 2011; Knochel et al., 2012; Yan et al., 2012
	Tractography	Manoach et al., 2007; Voineskos et al., 2010; Abdul-Rahman et al., 2011; de Weijer et al., 2011; Kunimatsu et al., 2012
Superior longitudinal fasciculus	VBM	Buchsbaum et al., 2006; Seok et al., 2007; Shergill et al., 2007; Skelly et al., 2008; Rotarska-Jagiela et al., 2009; Nakamura et al., 2012
	TBSS	Karlsgodt et al., 2008; Seal et al., 2008; Knochel et al., 2012
Arcuate fasciculus	VBM	Burns et al., 2003; Hubl et al., 2004; Kubicki et al., 2005; Munoz Maniega et al., 2008
	Tractography	Phillips et al., 2009; Catani et al., 2011; de Weijer et al., 2011
Inferior longitudinal fasciculus	VBM	Hubl et al., 2004; Shergill et al., 2007; Friedman et al., 2008; Skelly et al., 2008; Rotarska-Jagiela et al., 2009
	Tractography	Phillips et al., 2009
Occipital frontal fasciculi	VBM	Kubicki et al., 2005; Skelly et al., 2008; Rotarska-Jagiela et al., 2009; Nakamura et al., 2012
	TBSS	Seal et al., 2008; Knochel et al., 2012
Fornix	VBM	Kubicki et al., 2005
	Tractography	Abdul-Rahman et al., 2011; Kunimatsu et al., 2012
Corpus callosum	VBM	Foong et al., 2000; Hubl et al., 2004; Kubicki et al., 2005; Buchsbaum et al., 2006; Mori et al., 2007; Shergill et al., 2007; Rotarska-Jagiela et al., 2008, 2009; Kitis et al., 2011; Kong et al., 2011
	TBSS	Knochel et al., 2012; Roalf et al., 2013; Fujino et al., 2014
	Tractography	Kubicki et al., 2008; Kong et al., 2011
Anterior commissure	Tractography	Choi et al., 2011
Internal capsule	VBM	Kubicki et al., 2005; Buchsbaum et al., 2006; Munoz Maniega et al., 2008; Skelly et al., 2008; Sussmann et al., 2009; Nakamura et al., 2012; Levitt et al., 2012
	Tractography	Oh et al., 2009; Rosenberger et al., 2012
	TBSS	Seal et al., 2008; Knochel et al., 2012
Cortico-spinal tract	TBSS	Knochel et al., 2012
	Tractography	de Weijer et al., 2011
Corona radiata	VBM	Cui et al., 2011
	TBSS	Fujino et al., 2014
Middle cerebellar peduncles	VBM	Okugawa et al., 2004

As opposed to tract specific differences, several studies have reported widespread diffusion abnormalities in all tested regions (Lim et al., 1999; Minami et al., 2003; Reading et al., 2011; Schneiderman et al., 2011; Knochel et al., 2012), though not all DTI studies report positive findings when examining differences between schizophrenia patients and healthy controls. Two early studies showed no anisotropy differences in small samples of schizophrenia patients (Steel et al., 2001; Foong et al., 2002). A TBSS study found no differences in FA in a small sample of childhood-onset schizophrenia patients (Clark et al., 2012). Additionally, a recent tractography study reported no anisotropy differences in young adult patients with schizophrenia (Boos et al., 2012). A 2005 review of 15 DTI studies comparing schizophrenia patients to healthy controls concluded that, when the same ROI was investigated in different studies, as many failed to find a difference between groups as found one (Kanaan et al., 2005). Furthermore, a limitation of ROI studies is that the focus on one or a few specific structures neglects the rest of the brain. In an attempt to address this limitation, a brain-wide voxel wise meta-analysis and meta-regression analysis determined that decreased FA was restricted to interhemispheric fibers, anterior thalamic radiation, inferior longitudinal fasciculi, inferior frontal occipital fasciculi, cingulum and fornix (Bora et al., 2011). Overall, the above review of DTI findings in chronic patients with schizophrenia demonstrates that white matter abnormalities in chronic schizophrenia likely do exist. However, whether these abnormalities are localized to specific tracts, or spread throughout the brain is less clear. It appears that rather than specific tracts being affected, there is more likely an array of subtly altered networks distributed throughout the brain. Nevertheless, it could also be argued that the data suggest that the connectivity of frontal regions is particularly affected. This variability among studies with many white matter tracts seemingly affected, and difficulties in replicating findings is consistent with the conclusions of two recent reviews of DTI studies of schizophrenia (Melonakos et al., 2011; Fitzsimmons et al., 2013).

### First episode patients

Neuroimaging studies of patients with schizophrenia who are experiencing their first episode of psychosis can help to tease apart more definitively what is an intrinsic marker of the disorder, and what is a secondary effect of the illness. The main advantage of such studies is that the effects of long-term medication and chronicity can be ruled out as possible confounds. Concern about brain abnormalities being a result of treatment rather than being associated with the disease itself stem from studies that show that antipsychotic treatment is associated with smaller brain volumes in patients (Ho et al., 2011) and rodents (Vernon et al., 2011). The criteria for first episode subjects can be somewhat variable between studies but first episode patients are typically defined as being within 3 years of the onset of the first episode of psychosis. Some of these studies specifically examine patients that have never taken antipsychotic medications. DTI studies that examine first episode patients ask, are white matter abnormalities already present at illness onset?

In first episode patients evidence exists for tract specific white matter abnormalities in many of the same tracts identified in chronic patients, though findings are less consistent (Table 2). In contrast to tract specific differences, widespread DTI based changes throughout the brain have been reported in several studies of first episode schizophrenia subjects. In studies where patients have had minimal exposure to antipsychotic medications (i.e., weeks) VBM has revealed diffuse patterns of diffusion alterations in frontal, parietal and temporal lobes (Szeszko et al., 2005; Federspiel et al., 2006; Hao et al., 2006; Peters et al., 2009). A voxel wise meta analysis reported FA reductions in the white matter of the right deep frontal and left deep temporal lobes in first episode patients relative to healthy controls (Yao et al., 2013). Overall reduction in white matter tissue has been also been reported (Mendelsohn et al., 2006). In TBSS (Ruef et al., 2012; Lee et al., 2013) and tractography (Rathi et al., 2011) studies where patients were ill for 1 year or less on average and in one TBSS study of drug-naïve patients (Filippi et al., 2014) differences between patients and controls were present in white matter tracts throughout the brain. Free-water imaging—a recently developed methodology that can be applied to diffusion MRI data to distinguish neuroinflamation effects from axonal degeneration—has suggested that inflammation is widespread but axonal degeneration limited to focal areas in the frontal lobe white matter in first episode patients (Pasternak et al., 2012). In contrast, several other studies that have found no differences in all regions assessed in first episode samples of patients compared to healthy individuals, including those with minimal (Price et al., 2005, 2008; Friedman et al., 2008; Qiu et al., 2009; White et al., 2009) or no (Zou et al., 2008) antipsychotic medication use. Some studies found no FA abnormalities in first episode patients, but did identify possible abnormalities with other diffusion indices (Mendelsohn et al., 2006; Price et al., 2008; Chan et al., 2010; Moriya et al., 2010).

**Table 2:** **White matter tract alterations in first episode and medication naïve patients with schizophrenia**.

**White matter tract**	**Patient type**	**Comparison to healthy subjects**	**References**
Uncinate fasciculus	First episode	Difference	Price et al., 2008; Szeszko et al., 2008; Kawashima et al., 2009; Peters et al., 2009; Luck et al., 2011; Mandl et al., 2012; Lee et al., 2013
Superior longitudinal and arcuate fasciculi	First episode	Difference	Federspiel et al., 2006; Szeszko et al., 2008; Guo et al., 2012; Mandl et al., 2012
	Medication naive	Difference	Perez-Iglesias et al., 2010
Arcuate fasciulus	First episode	No difference	Peters et al., 2008
Cingulum bundle	First episode	Difference	Federspiel et al., 2006; Hao et al., 2006; Lee et al., 2013
	First episode	No difference	Peters et al., 2008; Kawashima et al., 2009; Luck et al., 2011
Genu of the corpus callosum	First episode	Difference	Price et al., 2007; Henze et al., 2012; Lee et al., 2013
	Medication naive	Difference	Perez-Iglesias et al., 2010
	First episode	No difference	Price et al., 2005; Cheung et al., 2008; Friedman et al., 2008; Peters et al., 2008; Gasparotti et al., 2009; Kong et al., 2011
	Medication naive	No difference	Cheung et al., 2008; Gasparotti et al., 2009
Splenium of the corpus callosum	First episode	Difference	Federspiel et al., 2006; Dekker et al., 2010
	Medication naive	Difference	Cheung et al., 2008; Gasparotti et al., 2009
	First episode	No difference	Price et al., 2005, 2007; Friedman et al., 2008; Peters et al., 2008
Internal capsule	First episode	Difference	Federspiel et al., 2006; Cheung et al., 2008; Perez-Iglesias et al., 2010; Guo et al., 2012; Lee et al., 2013
	First episode	No difference	Lee et al., 2012
	Medication naive	Difference	Perez-Iglesias et al., 2010
	Medication naive	No difference	Zou et al., 2008
Inferior longitudinal fasciculus	First episode	Difference	Chan et al., 2010
	Medication naive	Difference	Cheung et al., 2008; Perez-Iglesias et al., 2010; Liu et al., 2013
	First episode	No difference	Friedman et al., 2008
Occipital-frontal fasciculus	First episode	Difference	Szeszko et al., 2008; Lee et al., 2013
	Medication naive	Difference	Cheung et al., 2008; Perez-Iglesias et al., 2010; Liu et al., 2013
Optic radiations	First episode	Difference	Henze et al., 2012
Fornix	First episode	Difference	Guo et al., 2012; Lee et al., 2013

In summary, studies that identify differences between first episode patients and healthy controls suggest that DTI abnormalities are present in the early phase of schizophrenia. Studies that identify differences in medication naïve first episode patients support that these differences are not due to medication effects on the brain because they precede treatment. These results vary considerably between studies, currently no white matter tracts have been consistently identified though many studies have reported positive finding in the uncinate fasciculus. When taken together, the evidence suggests that DTI abnormalities in first-episode patients are less consistent than in chronic patients though this may be because some of these studies are underpowered, having small sample sizes because these patients are more difficult to recruit.

### High-risk subjects

In order to determine what brain abnormalities are present prior to illness onset, studies have examined people who are at high risk for developing schizophrenia. By following high-risk individuals and examining structural differences between those who go on to develop the disease and those who do not, this approach attempts to identify structural abnormalities that either predict future illness or act as indicators of resilience to developing schizophrenia. Different strategies have been employed to identify subjects at high risk for developing schizophrenia. In the Edinburgh high risk study, young asymptomatic subjects have at least two first- or second-degree relatives with a confirmed diagnosis of schizophrenia; more than half of these subjects developed psychotic symptoms within the first 5 years and 13% of this cohort went on to develop schizophrenia (Lawrie et al., 2008). The Melbourne ultra-high risk studies identified symptomatic, clinically compromised and help seeking individuals at risk of developing a florid psychosis, 30–40% of these subjects transition to psychosis in 12 months (Pantelis et al., 2007). Unlike studies in patients with schizophrenia, brain abnormalities in high-risk subjects cannot be attributed to secondary effects of the illness (i.e., neurotoxic effects of psychosis) or its treatment (i.e., medication effects). However, these high-risk subjects often already have attenuated psychotic symptoms, depressive symptoms, anxiety and other symptoms that may complicate interpretation of neuroimaging results. It should also be noted that differences in the nature of high-risk samples make it difficult to directly compare results between studies.

Two TBSS studies found altered DTI indices in high-risk subjects in the superior longitudinal fasciculus (Karlsgodt et al., 2009; Clemm von Hohenberg et al., 2014), a finding that was replicated in a voxel-based study that found intermediate FA values in high-risk subjects compared to a first episode group and controls in the this tract (Carletti et al., 2012). Differences in the corpus callosum, and posterior corona radiata have also been replicated (Carletti et al., 2012; Clemm von Hohenberg et al., 2014). Additionally, reduced FA has been found in left inferior longitudinal fasciculus, left inferior fronto-occipital fasciculus, right external capsule and right internal capsule in high-risk subjects (Carletti et al., 2012). A VBM study reported reduced FA in the superior frontal gyrus in high-risk subjects compared to healthy controls (Peters et al., 2009), though the same researchers failed to find differences between white matter tracts in these groups with tractography methods (Peters et al., 2008). In a longitudinal follow-up of the same sample, no differences were found between high-risk subjects who transitioned to psychosis and subjects that did not transition (Peters et al., 2010). Others did identify some DTI based differences in high-risk subjects who transitioned to psychosis compared to those who did not transition to psychosis, with lower FA lateral in the right putamen and in the left superior temporal lobe, and higher FA in the left medial temporal lobe (Bloemen et al., 2009). Finally, another study identified a reduction in FA in high-risk patients in left frontal white matter in those who developed psychosis, which was not evident in those who did not transition to psychosis (Carletti et al., 2012). These studies implicate alterations in these white matter regions early in the illness, which typically spans late adolescence and early adulthood. It is of note that the most consistent finding, changes in the superior longitudinal fasciculus, is a tract that is particularly dynamic in its development during this period in healthy subjects (Peters et al., 2012). These active changes that are occurring before and around the time of illness onset could potentially be ameliorated by early therapeutic interventions.

### Family studies

Another group of subjects who are of interest in brain imaging studies of schizophrenia are unaffected family members of patients with schizophrenia, most commonly siblings. Unaffected siblings share approximately 50% of the genome of the affected relative. DTI studies in schizophrenia patients that include a group of first-degree relatives attempt to characterize the extent to which differences in DTI metrics might reflect a genetic predisposition to schizophrenia through the identification of shared white matter abnormalities. In both schizophrenia patients and their first-degree relatives, reduced FA has been found in white matter associated in the left prefrontal cortex and in the medial temporal lobe, but not in the anterior cingulate cortex (Hao et al., 2009). One study focused on the frontal lobe and identified shared differences between patients and first-degree relatives in the medial frontal regions (Camchong et al., 2009). FA was shown to vary in accordance with relatedness to a patient in superficial white matter in the temporal and occipital lobes (Phillips et al., 2011). Additionally, a voxel-based study found reduced FA in first degree relatives compared to controls in inferior frontal, posterior cingulate, angular white matter, and increased FA in pontine tegmental, right superior frontal gyri and in the anterior cingulate (Hoptman et al., 2008). Tract specific analyses found differences in patients and relatives in the anterior limb of the internal capsule, but not the uncinate and the arcuate fasciculi (Munoz Maniega et al., 2008), and in the inferior fronto-occipital fasciculus, left inferior longitudinal fasciculus, and left temporal superior fasciculus (Clark et al., 2011). TBSS analysis in first-degree relatives of schizophrenia patients showed intermediate values between patients and controls in the inferior fronto-occipital fasciculus, superior longitudinal fasciculus, uncinate fasciculus, arcuate fasciculus and cingulum bundle (Knochel et al., 2012). FA alterations in these regions that are shared between patients and relatives may reflect a genetic liability of schizophrenia, though it will be important to see further replication of these results.

## Structural co-variance

Relationships between volumetric or cortical thickness measures in different regions have been used to investigate associations between brain regions. A straightforward version of this type of analysis has shown that schizophrenia is associated with differences in the bilateral symmetry of regions. Exaggeration of leftward thalamic asymmetry (Csernansky et al., 2004), reduced laterality in planum temporale (Ratnanather et al., 2013) and hippocampus (Kim et al., 2005) and reversal of the normal asymmetry of the inferior parietal cortex (Niznikiewicz et al., 2000; Buchanan et al., 2004) have been reported in schizophrenia patients. Examination of inter-regional structural co-variance can also be used to describe anatomical connectivity or coupling between brain regions, based on the principle that anatomically connected regions share common trophic factors and correlate in size (Alexander-Bloch et al., 2013). In these analyses correlations in interregional gray-matter volume or cortical thickness are computed in a group of subjects. Several studies have identified structural co-variance differences in schizophrenia patients compared to healthy control subjects with these methods (Table 3). Both strengthened (Breier et al., 1992; Wible et al., 1995, 2001; Mitelman et al., 2005b,c) and attenuated (Woodruff et al., 1997; Bullmore et al., 1998; Wright et al., 1999) co-variance between volumetric measures of the prefrontal and temporal cortex as well as stronger fronto-parietal relationships (Niznikiewicz et al., 2000; Buchanan et al., 2004; Abbs et al., 2011) have been described in patients. Additionally, altered cortico-thalamic (Portas et al., 1998; Mitelman et al., 2005a, 2006) and enhanced interhemisphereic prefrontal (Wheeler et al., 2014) structural co-variance have been reported in schizophrenia patients. Enhanced coupling within the default mode network and between default mode regions and the frontal cortex have been described in high-risk subjects (Bhojraj et al., 2010). Additionally, altered structural covariance within subgroups of schizophrenia patients has also been described. There is not a clear interpretation of the neural basis of schizophrenia-related changes in structural co-variance. Enhanced correlations may be related to overconnectivity, coordinated gray matter loss or a lack of developmental specificity, whereas diminished correlations between brain regions may suggest disconnectivity or localized degeneration.

**Table 3:** **Structural co-variance comparisons in patients with schizophrenia**.

**Study**	**Subjects**	**Relationships examined (# subregions)**	**Analysis**	**Results SCZ > HC^#^**	**Results HC > SCZ^#^**
Breier et al., 1992	44 SCZ	PFC (2) <-> temporal (1)	Interregional volume correlation	R PFC WM <-> R HPC/AMY	
	29 HC			
Wible et al., 1995	14 SCZ	PFC (2) <-> temporal (6)	Interregional volume correlation	L PFC GM<-> L ant HPC/AMY; L ant-PHG; L ant-STG L ant-PFC WM <->	L PFC GM <-> post-HPC/AMY
	15 HC			L ant-STG R PFC WM <-> R post-HPC/AMY	
				Among HPC/AMY, PHG, STG	
Woodruff et al., 1997	42 SCZ	PFC (2) <-> temporal (3)<-> CG (2)	Interregional volume correlation	DLPFC <-> VLPFC^*^	PFC <-> ant CG^*^
	43 HC				PFC <-> temporal^*^
					Post-CG <-> HPC^*^
Bullmore et al., 1998	35 SCZ	PFC (2) <-> temporal (3)<-> CG (2)	Interregional volume regression		PFC -> STG^*^
	35 HC				STG -> PFC ->HPC^*^
Portas et al., 1998	15 SCZ	THAL<->PFC; CG; striatum; HPC; AMY; STG	Interregional volume correlation	THAL <-> ventricle [neg]	THAL <-> L SFG [neg]
	15 HC			THAL <-> L frontal WM	
Wright et al., 1999	27 SCZ	Among: GM (92); ventricles (12)	Principle component	1st component (global GM <-> ventricles)^*^	
	37 HC			4th component (frontal<->temporal)^*^	
Niznikiewicz et al., 2000	15 SCZ	Parietal (3)<-> frontal (4); temporal (4)	Interregional volume correlation	L IPL <-> PFC^*^	L post-CG <-> L OFC; R OFC; R SFG
	14 HC			L post-CG <-> R IFG	
				L and R IPL <-> L ant STG	
Wible et al., 2001	17 SCZ	PFC (2) <-> temporal (3)	Interregional volume correlation	R PFC GM <-> R post-HPC/AMY	
	17 HC				
Buchanan et al., 2004	44 SCZ	Among: PFC (4); STG; IPL (2)	Interregional volume correlation	L and R IFG <-> L and R AG^*^	
	34 HC				
Mitelman et al., 2005a	106 SCZ	THAL(5) <-> Broadmann areas (39)	Interregional volume correlation		R THAL<-> R PFC; R MTG^*^
	42 HC				L THAL<-> L PFC; L CG; L post-parietal; occipital^*^
Mitelman et al., 2005b	106 SCZ	Frontal (11) <-> other Broadmann areas (39)	Interregional volume correlation	Frontal <->temporal; parietal; occipital	Among frontal^*^
	42 HC				
Mitelman et al., 2005c	106 SCZ	Temporal (12) <-> other Broadmann areas (39)	Interregional volume correlation	Frontal <-> temporal^*^	Frontal <-> temporal [neg]^*^
	42 HC				R temporal pole <-> other temporal^*^
Mitelman et al., 2006	41 SCZ (unmedicated)	THAL(3) <-> Broadmann areas (39)	Interregional volume correlation	Pulvinar <-> DLPFC; temporal [neg]^*^	R pulvinar <-> R OFC; R occipital^*^
	59 HC				Centromedian nucleus <-> DLPFC^*^
Bhojraj et al., 2010	64 SCZ offspring	DLPFC <-> PCUN; IPL; lat-temporal; ant-CG; post-CG; med-PFC	Interregional volume correlation	L DLPFC <-> L lat-temporal^*^	
	80 HC	Among PCUN; IPL; lat-temporal; ant CG; post-CG; med-PFC		R DLPFC <-> R ant-CG^*^	
				L ant-CG <->L lat-temporal; L post-CG; L MPFC^*^	
				R IPL <-> R lat-temporal; R post-CG^*^	
				R ant-CG <-> R lat-temporal; R IPL^*^	
Abbs et al., 2011	88 SCZ	Among PFC; IPL; ant CG; PHG; HPC	Interregional volume correlation	HPC<-> ant-CG [neg] (in F only)^*^	
	48 HC			IPL <-> ant-CG (in F only)^*^	
Wheeler et al., 2014	54 SCZ	L DLPFC <-> cortex (81, 923 vertices)	Interregional cortical thickness correlation	L DLPFC <-> R DLPFC; R VMPFC^*^	
	68 HC				

# Results reported as significant positive correlations in one group and not the other unless indicated by [neg] in which case there are significant negative correlations in one group but not the other. Significant differences between groups are indicated by ^*^.

*SCZ, schizophrenia; HC, healthy control; ant, anterior; post, posterior; med, medial; lat, lateral; L, left; R, right; F, female*.

*Symbol: <->, between*.

*Brain regions: AG, angular gyrus; AMY, amygdala; CG, cingulate gyrus; DLPFC, dorsal lateral prefrontal cortex; GM, gray matter; HPC, hippocampus; IFG, inferior frontal gyrus; IPL, inferior parietal; Ins, Insular cortex; ITG, inferior temporal gyrus; MTG, middle temporal gyrus; OFC, orbital frontal cortex; PCUN, precuneus; PFC, prefrontal cortex; PHG. parahippocampal gyrus; SFG, superior frontal gyrus; STG, superior temporal gyrus; THAL, thalamus; VLPFC, ventral lateral prefrontal cortex; VMPFC, ventral medial prefrontal cortex; WM, white matter*.

## Network analyses

**Figure 2:**
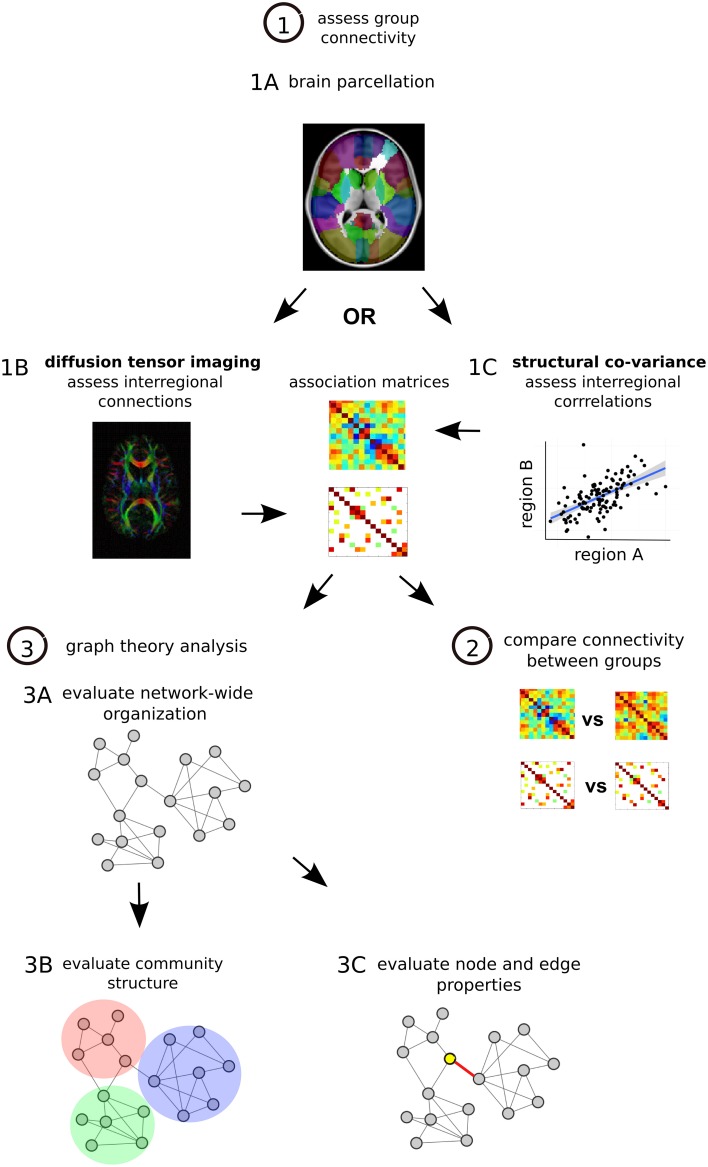
**Structural network analysis steps. (1)** Assess group connectivity. Perform MRI imaging and parcellate the brain, many different methods can be applied (Craddock et al., 2013) **(1A)**. Quantify DTI-based structural connectivity or morphometry-based structural co-variance throughout the brain. For DTI the presence and strength of interregional connections are assessed, one matrix is generated for each subject and then a group average is calculated **(1B)**. For structural co-variance analysis between-subject correlations in morphology are calculated in each group **(1C)**. In each case associations are described with an association matrix whose rows and columns correspond to different brain regions. **(2)** Compare connectivity between groups. Statistical comparison can be done on a connection by connection basis with permutation testing or with Network Based Statistics (NBS), a statistical approach that is able to identify altered sub-networks while controlling the family wise error rate associated with testing for differences between a large number of connections (Zalesky et al., 2010). **(3)** Graph theory analysis. Convert matrices to networks with collections of nodes (brain regions) interconnected by edges (connections) (Bullmore and Sporns, 2009). Edges describe the degree of anatomical connectivity or coupling between network nodes and can be either weighted according to the strength of measured connectivity or unweighted and binary. Once networks are established, three basic types of graph theory measures can be assessed. First graph theory metrics can assess network-wide integration (characteristic path length, global efficiency) and segregation (mean clustering coefficient, mean local efficiency) as well as characterize network architecture (mean degree, degree distribution, mean connectivity, assortativity, hierarchy, small-world organization) **(3A)**. Second, networks can be assessed for their modular structure, identifying distinct communities of nodes and connections that cluster together **(3B)**. Third, at the level of individual regions and connections, nodes and edges can be assessed for centrality, which is thought to reflect the potential for enabling efficient communication in the network. These centrality measures are based on number of connections (degree) and positioning within the network (betweenness, eigenvector centrality, closeness). Hub regions in the brain that are thought to play more integral roles in network function due to their central positioning can be described with these centrality metrics (Rubinov and Bullmore, 2013) **(3C)**.

Network-based analyses may provide a characteristic neural signature of illness in schizophrenia. Recently, methods of measuring structural brain connectivity have been used to comprehensively map the large-scale architecture of connectivity in the brain by quantifying pair-wise interactions between regions throughout the brain (Sporns, 2011). Graph theory—a branch of mathematics that is used to study and describe all types of networks—can be used to compute various organizational properties of brain-wide connectivity^193^ (Figure 2). A large appeal graph theory rests in its ability to provide relatively succinct, characterizations of regional and whole-brain disturbances in brain network connectivity and topology^195^.

Altered connectivity and brain network topology has been described in the brains of schizophrenia patients (Table 4). Structural connectomic studies have identified altered connectivity within sub-networks in chronic patients (Skudlarski et al., 2010; Zalesky et al., 2011; Collin et al., 2013), as well as first episode medication naïve patients (Zhang et al., 2014) and siblings of patients (Collin et al., 2014). Graph theoretical analyses at the whole network level have predominantly identified equivalent small-world organization (Bassett et al., 2008; van den Heuvel et al., 2010, 2013; Zalesky et al., 2011; Wang et al., 2012; Zhang et al., 2014) and network inefficiency with reduced integration between regions in chronic patients (Bassett et al., 2008; van den Heuvel et al., 2010; Zalesky et al., 2011; Wang et al., 2012; Zhang et al., 2012b; Ottet et al., 2013), first episode medication naïve patients (Zhang et al., 2014), unaffected siblings (Collin et al., 2014) and high-risk infants (Shi et al., 2012). At the regional level network studies have provided strong evidence for reorganization of network hubs in the brains of schizophrenia patients (Rubinov and Bullmore, 2013), commonly with a reduction of these regions localized to the frontal cortex and limbic areas (Bassett et al., 2008; van den Heuvel et al., 2010; Shi et al., 2012; Wang et al., 2012; Zhang et al., 2012b; Ottet et al., 2013; Collin et al., 2014) including in the study of first episode medication naïve patients (Zhang et al., 2014). Additionally, reduced connectivity between frontal and parietal hubs has been described in schizophrenia patients and their unaffected siblings (van den Heuvel et al., 2013; Collin et al., 2014). Though DTI-based structural connectivity and morphometry-based structural co-variance are related to each other (Gong et al., 2012), interpretation of results from these two types of studies can be distinct. Despite methodological differences many of the above-described global results from network analyses in schizophrenia patients are consistent. However, localized results of altered network properties in specified brain systems are much more varied between studies.

**Table 4:** **Network analysis of structural connectivity in schizophrenia**.

**Study**	**Network type**	**Subjects**	**Node definition**	**Edge definition**	**Analysis**	Global results^*^	Local Results^*^	Hubs^*^
Bassett et al., 2008	Structural correlation (1.5 T)	203 SCZ	108 regions Pick atlas	Binary, correlation between region volumes	Graph theory	=SW, K distribution	↓hierarchy, ↑mean C distance in multimodal network	↓PFC hubs
		259 HC					≠ CC, K and B in PreM, PFC, OFC, ITG, MTG, CG, Ins	↑ITG, Ins, CG hubs
Zhang et al., 2012b	Structural correlation (1.5 T)	101 SCZ	78 regions AAL atlas	Binary, correlation between regional cortical thickness	Graph theory	↑PL, CC	↓B mainly in the association cortices	↑# of hubs
		101 HC					↑B in the primary and paralimbic cortices	↓association cortex hubs
								↑primary, paralimbic hubs
Collin et al., 2013	Structural correlation (1.5T)	146 SCZ	(1) 12 lobes	Binary, correlation between region volumes	C compare (lobes)	n/a	↓C frontal <-> subcortical	n/a
		122 HC	(2) 82 regions Freesurfer		NBS (regions)		↑C temporal <-> subcortical; L frontal <-> R frontal; frontal <-> limbic impaired networks:1)	
							PFC, temporal, occipital, parietal, limbic 2) SMG, Post-CG, CG, subcortical (NBS)	
Shi et al., 2012	Structural correlation (3T)	26 high risk neonates^$^	90 regions adapted AAL atlas	Binary, correlation between region volumes	Graph theory	↓Eglob, inter-module C	n/a	=frontal, temporal hubs
		26 HC neonates				↑Eloc, PL, CC, C distance		↑occipital hubs
								↓parietal, subcortical hubs
Shi et al., 2012	DTI white matter (3T, 6 dir)	26 high risk neonates	90 regions adapted AAL atlas	Binary, nodes linked if sufficient # of streamlines	Graph theory	=Eglob, Eloc, C distance	↓number of fibers in 4 right subcortical <-> cortical and 2 occipital <-> limbic Cs	=frontal, temporal, insula, limbic hubs
		26 HC neonates			C compare			↑occipital hubs
								↓parietal, subcortical hubs
van den Heuvel et al., 2010	DTI white matter (1.5T, 32dir)	40 SCZ	108 regions AAL atlas	Weighted by average MTR -nodes linked if sufficient # of streamlines	Graph theory	=SW, C strength, PL, CC	↑PL of frontal and temporal regions	↓B in frontal hubs
		40 HC					↓CC in HPC and paracentral lobule	
Skudlarski et al., 2010	DTI White matter (3T, 12dir)	27 SCZ	6000 K means clustering	Weighted by # of streamlines	C compare	Decoupling between anatomical and functional C	↓C in in default mode and task positive networks	n/a
		27 HC						
Zalesky et al., 2011	DTI white matter (1.5T, 64dir)	74 SCZ	82 regions AAL atlas	Binary, nodes linked if sufficient # of streamlines	Graph theory	=SW	↓C in AG <-> R MTG; R SFG <-> L SFG; L IFG <-> L Ins	n/a
		32 HC			C compare	↓K, Eglob	↓C in network of medial frontal regions connected to parietal and occipital lobes (NBS)	
					NBS			
Wang et al., 2012	DTI white matter (1.5T, 13dir)	79 SCZ	90 regions AAL atlas	Weighted by # of streamlines	Graph theory	↓Eglob	↓regional Eglob in frontal associative cortices, paralimbic/limbic and subcortical regions	=association cortex, paralimbic/ limbic hubs
		96 HC				=Eloc, SW		
van den Heuvel et al., 2013	DTI white matter (3T, 32dir)	48 SCZ	82 regions	Weighted by # of streamlines	Graph theory	↓RC organization, C strength, Eglob, CC	↓RC organization most pronounced in cortical networks	=RC members: PCUN, SFG, SPG, Ins
		45 HC	Freesurfer			↑M	↓density of RC Cs	
						=SW		
Collin et al., 2014	DTI white matter (1.5T, 32dir)	40 SCZ	68 cortical regions	Weighted by # of streamlines	Graph theory	↓C strength, Eglob (HC>SCZ)	↓C strength: SFG, ITG ↓Eglob: ACG, SFG, and PreCG	≠C and CC over-represented in RC members
		54 SCZ siblings	Freesurfer		NBS	↓CC (HC>siblings>SCZ)	↓CC: SFG, ACG, OFG, PreCG and Ins	↓C over- represented in RC members (NBS)
		51 HC					↓RC and local C strength (HC>siblings>SCZ)	
Ottet et al., 2013	DTI white matter (3T, 30dir)	46 22q11.2DS	82 regions	Binary, nodes linked if sufficient # of streamlines	graph theory	↑PL	↓centrality in 16 out of 65 nonhubs (25%)	↓centrality in 10 out of 17 hubs (58%): HPC, SPG and PreCG, MFG, SFG, PCUN, THAL
		48 HC	Freesurfer			↓Eglob, K		
						=CC		
Zhang et al., 2014	DTI white matter (1.5T, 15dir)	30 FE-SCZ	90 regions AAL atlas	Weighted by # of streamlines	graph theory	=SW	↓Eglob and K in sensorimotor, basal ganglia, and limbic-visual systems	=hubs: PCUN, FG, MTG, ITG, SPG, post-CG
		34 HC			NBS	↓C strength, Eglob, K	↓C in subnetwork of frontal, parietal, occipital and subcortical regions (NBS)	↓hubs: MFG, post-CG and PreCG
						↑PL		

* Results reported in patient groups relative to controls unless otherwise indicated;

$ *High risk neonates have mothers with schizophrenia*.

*SCZ, schizophrenia; SA, schizoaffective disorder; SF, schizophreniform disorder; FE-SCZ, first episode, medication naïve schizophrenia; HC, healthy control; 22q11.2DS - 22q11.2 deletion syndrome; NBS, network based statistics; MTR, magnetization transfer ratio; AAL, Automated Anatomical Labeling atlas (Tzourio-Mazoyer et al., 2002); LPBA40, LONI Probabilistic Brain Atlas (Shattuck et al., 2008)*.

*Graph theory metrics: SW, small world organization; CC, clustering coefficient; PL, mean path length; K, degree; B, betweenness centrality; Eglob, global efficiency; Eloc, local efficiency; C, connectivity; M, modularity; RC, rich club*.

*Symbols: <->, between; #, number; ↑, increased; ↓, decreased; =, equivalent; ≠, different*.

*Brain regions: AG, angular gyrus; CG, cingulate gyrus; HPC, hippocampus; IFG, inferior frontal gyrus; Ins, Insular cortex; ITG, inferior temporal gyrus; MFG, middle frontal gyrus; MTG, middle temporal gyrus; OFC, orbital frontal cortex; PCUN, precuneus; PFC, prefrontal cortex; PreCG, precentral gyrus; PreM, premotor cortex; SFG, superior frontal gyrus; SPG, superior parietal gyrus; THAL, thalamus*.

## Heterogeneity of findings

Neuroimaging investigations in schizophrenia have produced a large body of evidence for structural connectivity abnormalities associated with the illness. It is clear from this review of the literature that there is considerable variability among white matter tract investigations in chronic patients as well as in first episode patients, individuals at high risk to develop the illness and first-degree family members, as well as in structural co-variance and network-based investigations. There are several likely contributing factors to this heterogeneity. First, methodological differences exist within imaging modalities, and the same dataset analyzed with different techniques may produce different results and therefore different conclusions. Second, a large contributing factor to the heterogeneity of results is due to the fact that the clinical presentation of schizophrenia itself is very heterogeneous. A number of studies that are presented here combine a range of schizophrenia diagnostic subtypes within their experimental cohort, including schizoaffective disorder, schizophreniform disorder and schizotypal personality disorder. Even within the diagnosis of schizophrenia, different subtypes of the illness have been proposed, such as the deficit subtype that describes patients with primary and enduring negative symptoms. The inclusion of different diagnostic categories within one experimental cohort makes any subsequent results inherently more difficult to interpret. Third, the causal mechanisms underlying schizophrenia are themselves heterogeneous, so brain abnormalities likely vary according to these and other genetic and environmental factors, as well as the developmental stage in which these occur. Finally, there is a large difference in moderator variables across studies such as age of onset, gender, chronicity, medication and parental socio-economic status, complicating the direct comparability of one study to another. For example, differences in FA have been reported between smoking and nonsmoking subjects (Cullen et al., 2012), which could potentially confound results if this detail is not accounted for in schizophrenia and control populations that are being compared. Moreover, it should be noted that identified morphological alterations are likely not specific to the diagnosis of schizophrenia. For example, substantial overlap in the localization of white matter FA reductions exists in schizophrenia and bipolar disorder (Sussmann et al., 2009). There are a number of other potential factors, such as variations in genes, which could lead to inconstant findings in these studies (Lett et al., 2013).

To address the influence of clinical heterogeneity of schizophrenia on brain connectivity, brain imaging metrics have been examined in relation to symptoms. Negative symptom severity has been associated with lower FA in the bilateral uncinate fasciculus and inferior frontal white matter (Wolkin et al., 2003; Szeszko et al., 2008). When comparing schizophrenia patients with the deficit syndrome to patients without the deficit syndrome and control subjects, deficit patients have been shown to have distinct DTI-based measures in the right inferior longitudinal fasciculus, right arcuate fasciculus, left uncinate fasciculus and right superior longitudinal fasciculus (Rowland et al., 2009; Kitis et al., 2012; Voineskos et al., 2013). One first episode study reported a negative correlation between FA values in the white matter adjacent to the right lateral ventricle and negative symptom scores and a positive correlation of FA values with positive symptom scores in the same brain region (Moriya et al., 2010). Similarly, increased FA in several regions was related to more severe positive symptoms as well as longer duration of untreated psychosis in a recent study (Filippi et al., 2014). Auditory hallucinations have been positively related to FA in the anterior corpus callosum, cingulum and superior longitudinal fasciculus (Hubl et al., 2004; Seok et al., 2007; Shergill et al., 2007). FA in the inferior fronto-occipital fasciculus (Szeszko et al., 2008) and structural co-variance between the frontal and temporal cortex (Modinos et al., 2009) have been shown to correlate with greater severity of hallucinations. Lower FA values in inferior fronto-occipital fasciculus, left superior longitudinal fasciculus and left uncinate fasciculus were significantly associated with higher levels of positive symptoms and with duration of illness (Knochel et al., 2012). Furthermore, in first-episode schizophrenia patients, positive symptoms correlated positively with FA scores in white matter associated with the right frontal lobe, left anterior cingulate gyrus, left superior temporal gyrus, right middle temporal gyrus, right middle cingulate gyrus, and left cuneus (Cheung et al., 2011).

Brain connectivity has also been related to cognitive impairment, functional outcome and other characteristics in schizophrenia patients. Working memory performance has been associated with altered FA in the cingulate fasciculi (Nestor et al., 2010) and left superior longitudinal fasciculus (Karlsgodt et al., 2008), as well as cortical thickness co-variance between the posterior cingulate gyrus and ventral medial prefrontal cortex (Wheeler et al., 2014). Reduced FA in the left inferior longitudinal fasciculus and left inferior fronto-occipital fasciculus has been correlated with reduced processing speed, as well as verbal learning and visual learning abilities (Liu et al., 2013). Additionally, greater declines in intelligence correlated with reduced cingulum white matter FA (Nestor et al., 2013) and FA of several white matter tracts has been related to cognitive performance across a number of domains in people with schizophrenia (Voineskos et al., 2012). Studies have shown that patients with poor outcome compared to good-outcome had more pronounced FA reductions in the posterior corpus callosum and fronto-occipital fasciculus (Mitelman et al., 2007), as well as altered structural co-variance patterns (Mitelman et al., 2005a,b,c). Reduced FA in the inferior longitudinal fasciculus predicted social functioning at follow-up in high-risk subjects (Karlsgodt et al., 2009), and inferior frontal white matter microstructure was associated with impulsivity and aggression in men with schizophrenia (Hoptman et al., 2002). Furthermore, structural covariance relationships differed by sex in patients with schizophrenia (Abbs et al., 2011).

## Summary and conclusions

These studies provide insight into structural connectivity based brain correlates of schizophrenia. Widespread alterations in connectivity are present in the brains of chronic and first episode schizophrenia patients and there is some evidence that patterns of reduced connectivity cut across the different stages of the disorder, including those with an increased risk of developing the illness. Less consistency in first episode, high-risk studies may be because neuroanatomical differences are subtler at the time of illness onset, or because they only appear after a longer time-course in schizophrenia and could possibly be due to treatment. Evidence suggests that differences in the brains of immediate family members are intermediate to patients and control subjects, pointing to a genetic contribution to brain alterations. The diffuse nature of brain abnormalities implies that brain disruption may be related to altered network function. There is a large amount of heterogeneity in structural connectivity findings, some of which may be explained by associations between neuroimaging measures, and symptoms, cognitive performance and outcome.

Many questions about imaging brain connectivity in schizophrenia remain to be answered. Significantly, what are the microscopic neuroanatomical substrates of these changes that are detected with DTI? Reductions in FA may reflect myelin structure, myelin content or axon diameter. These issues may be addressed with advanced imaging or post-mortem studies. Technical advancements in MRI imaging, particularly novel diffusion imaging approaches have the capability to delve further into the underlying architectural changes in the brain. One limitation of current DTI approaches is that each voxel cannot be assumed to contain a single, coherently oriented bundle of white matter axons. Acquisition protocols, such as high angular resolution diffusion-weighted imaging (HARDI) (Tuch et al., 2002) and diffusion spectrum imaging (DSI) (Wedeen et al., 2005, 2008), extract more information from the diffusion-weighted signal and can resolve crossing fibers within voxels. A second limitation is that the DTI model assumes a single pool of diffusing water to calculate FA, which does not describe the diffusion decay in white matter. This has been addressed by using a multi-b-value diffusion imaging acquisition protocol that allows for the use of a permeability diffusivity model that estimates the permeability of cellular membranes (Kochunov et al., 2013). Another analytic method separates diffusion properties of brain tissue from surrounding free water permitting computation of the free water volume, which contributes to the architecture surrounding neuronal bundles, and may indicate specific anatomical processes such as neuroinflammation and axonal degeneration (Pasternak et al., 2009). Additionally, neurite orientation dispersion and density imaging (NODDI) allows for the estimation of the microstructural complexity of dendrites and axons by adopting a model-based strategy which relates the signals from diffusion MRI to geometric models of tissue microstructure (Zhang et al., 2012a). Increased sharing of data will allow for increased sample sizes, which will increase the power of statistical analyses and allow for replication of results, a strategy that will greatly benefit the field. With the continued advancement of imaging and analysis techniques, the neural substrates of schizophrenia are certain to be further elucidated. The identification of affected circuits will guide the application of therapeutic interventions to treat this devastating disorder.

### Conflict of interest statement

The authors declare that the research was conducted in the absence of any commercial or financial relationships that could be construed as a potential conflict of interest.
